# Mental abilities and performance efficacy under a simulated 480-m helium–oxygen saturation diving

**DOI:** 10.3389/fpsyg.2015.00979

**Published:** 2015-07-09

**Authors:** Gonglin Hou, Youlan Zhang, Na Zhao, Ruiyong Chen, Weibing Xiao, Hao Yu, Jiachun Wang, Ti-Fei Yuan

**Affiliations:** ^1^Institute of Cognitive Neuroscience and Department of Psychology, Zhejiang Sci-Tech UniversityHangzhou, China; ^2^Institute of Naval MedicineShanghai, China; ^3^School of Psychology, Nanjing Normal UniversityNanjing, China

**Keywords:** saturation diving, spatial memory, mental rotation, grip strength, hand–eye coordination, cognition, helium–oxygen, stress

## Abstract

Stress in extreme environment severely disrupts human physiology and mental abilities. The present study investigated the cognition and performance efficacy of four divers during a simulated 480 meters helium–oxygen saturation diving. We analyzed the spatial memory, 2D/3D mental rotation functioning, grip strength, and hand–eye coordination ability in four divers during the 0–480 m compression and decompression processes of the simulated diving. The results showed that except for its mild decrease on grip strength, the high atmosphere pressure condition significantly impaired the hand–eye coordination (especially above 300 m), the reaction time and correct rate of mental rotation, as well as the spatial memory (especially as 410 m), showing high individual variability. We conclude that the human cognition and performance efficacy are significantly affected during deep water saturation diving.

## Introduction

Stress in extreme environments severely affects the body and mind. For instance, during diving the physical stress (underwater pressure) increases with the water depth (11 atm at 100 m, and 48.6 atm at 480 m, for example; Brubakk et al., 2014). This creates inflammation signaling (Matsuo et al., 2000; Krog et al., 2010), oxidative stress load (Ikeda et al., 2004), extreme tiring experience of body, stress hormone secretion (Hirayanagi et al., 2003), sleep disruption (Nagashima et al., 2002), and the psychological stress in mind (Curley et al., 1979; Biersner et al., 1984), resulting in alterations of divers’ mood, cognition and performance efficacy, which are critical for diving work.

Previous studies reported different results on relevance between deep water diving (more than 300 m) and mental abilities, potentially due to the limited availability of subjects. In one study, it is found that 360 m helium–oxygen saturation diving did not affect the finger flexibility of divers (Hamilton, 1976); while another study reported increased finger tremors at 485 m diving (especially after 304 m) in six divers (Berghage et al., 1975). Other studies reported significant variability across individuals concerning the finger tremors (300 m), which is reduced in professional divers (Yamasaki et al., 1985). In addition, it is known that the spatial visual capacity is disrupted by high pressure (300–549 m; Lewis and Baddeley, 1981), and that the cognition is affected (Carter, 1979). Such mixed results indicate that depth and gas mixture are probably not the only factors. When exposed to high pressure, divers suffer from not only physical pressure but also psychological stress, such as anxiety, fear, and rapture during compression, underwater operation, and decompression.

The previous Asian record of saturation dive was 450 m Japan, yet the psychological changes during the dive have not been investigated. In addition, few studies systemically investigated the spatial memory, 2D/3D mental rotation, grip strength, and hand–eye coordination abilities during deep water saturation dive. We therefore hypothesized that the deep water diving might impair these aspects of mental abilities and performance efficacy when above certain levels of atmosphere pressure. With present study, we investigated these aspects during the compression and decompression processes of this 480 m (the new Asian record) saturation dive.

## Materials and Methods

### Subjects

Four male professional divers (right-handed, 27–32 years old, average at 30.3 ± 2.22) were recruited for this study. They passed strict physical and psychological examinations and were healthy.

The study is approved by ethic committee of human study in Zhejiang Sci-Tech University and all subjects provided written consent for experimental procedures. The procedures followed the guidelines of human research from ethic committee in Zhejiang Sci-tech University and Nanjing Normal University.

### Experimental Methods

For grip strength measurement, CWJ-1 grip scale (range 0–100 kg) was provided by Service center from Sports department of China government. The measurement was performed three times for each hand, and the maximum value was taken (kg). The subjects recorded the values for each other.

For hand–eye coordination experiment, 4-hole buttons (button diameter 12.5 mm, hole diameter 1.5 mm) were used (three holes blocked). An 80 cm long 1 mm diameter cotton wire was used. In the test, the subjects were asked to thread the wire through the remaining button hole to connect as many buttons as possible in 1 min. The test was repeated three times and the average was taken.

**FIGURE 1:**
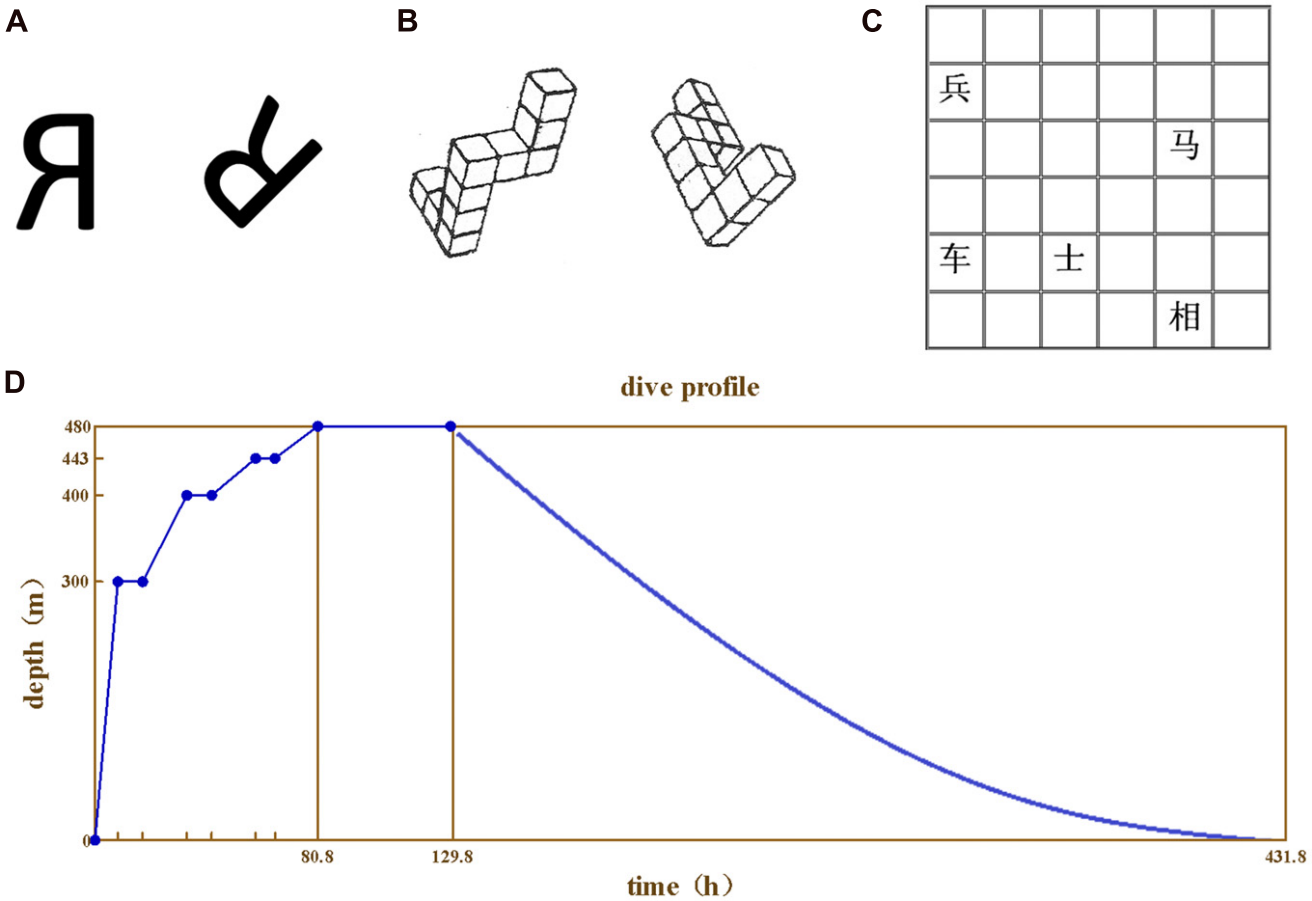
**The test materials and the compression procedure. (A)** 2D mental rotation, **(B)** 3D mental rotation, **(C)** Spatial memory test, **(D)** The compression and depression procedures.

For 2D mental rotation test, two “R” characters rotated at 45°/90°/135°/180° (five questions for each degree, 20 questions in total) were used (**Figure 1A**). The subjects were asked to judge whether the two “R” characters were exactly the same or in mirror image. The reaction time and correct rate were recorded.

For 3D mental rotation test, two 3D images were used for each question (20 questions in total) as previously described (Shepard and Metzler, 1971; **Figure 1B**). The subjects were asked to judge whether the two images could merge to each other. The reaction time and correct rate were recorded.

For spatial memory test, the 6 × 6 grids paper printed with Chinese chess characters were used (**Figure 1C**). Each paper was printed with five characters at random grids. The subjects were asked to remember the name and the place of the five characters on the paper in 30 s. The experiment was repeated for five times with different papers each time. The correct rate was recorded.

### Simulation of Saturation Diving

The simulation was conducted at the diver living chamber of 500 m saturation diving system at Institute of Naval medicine, Shanghai, China. The chamber has an internal diameter of 2.2 m and length 5.5 m and living volume 19.5 m^3^. The chamber is filled with helium–oxygen, temperature 31 ± 1°, moisture 50–70%, noise lower than 75 dB and light intensity 50–100 lx.

The study was divided into predive, compression, stay, decompression, and postdive phases. For all tests presented above, the divers were trained repeatedly to reach a stable baseline, and the last score was taken as baseline. During the compression phase, the atmosphere pressure supplied at 0–10 m was 21% helium–oxygen, and 10–480 m with pure helium. The rate of pressure increase was at 0.6 m/min for 0–300 m, 0.1 m/min for 301–400 m, and 0.04 m/min for 401–480 m. The subjects stayed at 300, 400, and 443 m for 7–9 h each. The compression and depression procedures were described in **Figure 1D**.

For cognition tests (spatial memory and mental rotation), the data was collected at 0 m before, 150/350/410/463 m during compression, the early/late phase of 480 m stay, 400/308/230/154/83/25 m during decompression, 3 days and 1 month after the test at 0 m.

For grip strength test, the data was collected at 0 m before, 150/270/300/400 m during compression, the early/late phase of 480 m stay, 446/398/357/314/274/230/192/154/119/85/54/25 m during decompression, 3 days and 1 month after the test at 0 m.

For hand–eye coordination test, the data was collected at 0 m before, 150/270/300/400/463 m during compression, the early/late phase of 480 m stay, 400/308/230/154/83/25 m during decompression, 3 days and 1 month after the test at 0 m.

### Statistics

The data is analyzed with SPSS 17.0 software (Chicago, IL, USA) for database building. The results were tested with analyses of variances and LSD *post hoc* test. The data was normalized to baseline point (100%) for better visualization and understanding, and to decrease the effects of individual variability at a small *n*.

## Results

### Mental Abilities

#### 2D Mental Rotation

**FIGURE 2:**
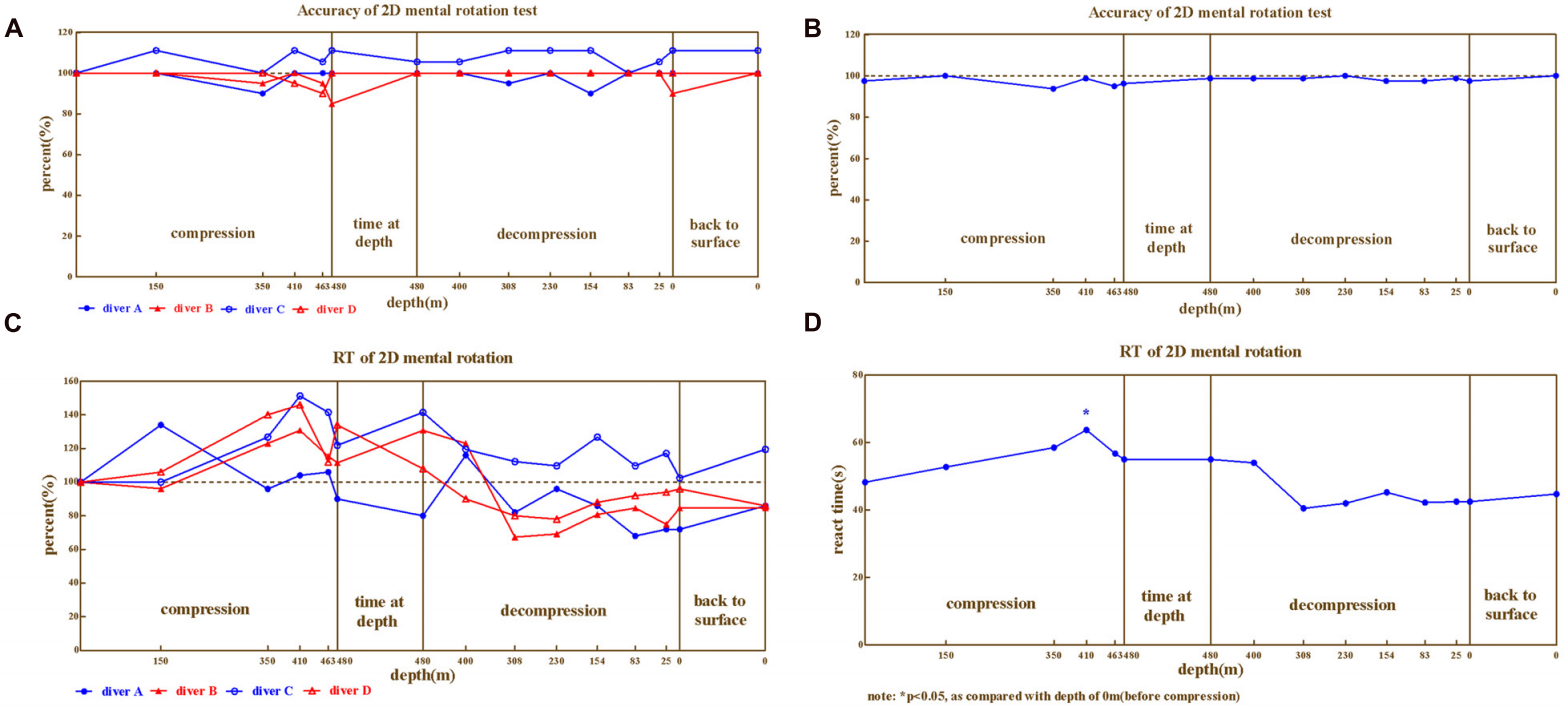
**The results from 2D mental rotation. (A)** Correct rate for four divers, **(B)** Correct rate on Average, **(C)** Reaction time for four divers, **(D)** Reaction time on average.

In 2D mental rotation, the correct rates for four divers (**Figure 2A**) and average (**Figure 2B**) exhibited mild changes during the compression and decompression phases (ranged 85%∼111.11% of baseline). While the reaction times of four divers (**Figure 2C**) and the average (**Figure 2D**) exhibited greater fluctuations (ranged 67.31%∼151.22% of baseline). There was also noticeable variability among individual divers. The correct rates were worse than baseline for diver A, B, and D (ranged 85%∼100% of baseline), but better for diver C (ranged 100∼111.11% of baseline). The variability of reaction time of the four divers was much larger. Reaction times were negatively affected as compression continued until the depth of 410 m for diver B, C, and D, but not for diver A (except the depth of 150 m in compression and 400 m in decompression).

Repeated variance analyses revealed that the depth had a significant effect on reaction time [*F*_(14,42)_ = 4.226, *P* < 0.05, $\eta_{p}^{2}$ = 0.585], but not on the correct rate [*F*_(14,42)_ = 0.864, *P* > 0.05, $\eta_{p}^{2}$ = 0.224]. During the compression phase, the reaction time continuously increased until 410 m (LSD *post hoc* test *P* < 0.01 when compared to predive).

We further compared the 2D mental rotation ability across different phases of the experiment (predive, compression, stay, decompression, 3 days, and 1 month after the dive; **Table 1**). Repeated variance analyses revealed that the phase of the experiment had a significant effect on the reaction time [*F*_(5,15)_ = 7.09, *P* < 0.05, $\eta_{p}^{2}$ = 0.703], but not on the correct rate [*F*_(5,15)_ = 5.05, *P* > 0.05, $\eta_{p}^{2}$ = 0.138]. LSD *post hoc* test revealed that the reaction times of the four divers significantly increased in the compression phase (when compared to the baseline, *P* < 0.05) and returned to the baseline levels during the decompression phase (*P* > 0.05 when compared to predive).

**Table 1:** 2D mental rotation ability across different phases of the experiment (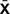 ± SD, *n* = 4).

Phase	Reaction time(s)	Correct rate(%)
Predive	48.25 ± 4.92	97.50 ± 5.00
Compression	57.94 ± 4.58	96.88 ± 0.72
Stay	55.00 ± 9.16	97.50 ± 3.54
Decompression	44.42 ± 2.06	98.54 ± 1.72
3 days after	42.50 ± 5.00	97.50 ± 5.00
1 month after	44.75 ± 2.87	100 ± 0.00

#### 3D Mental Rotation

**FIGURE 3:**
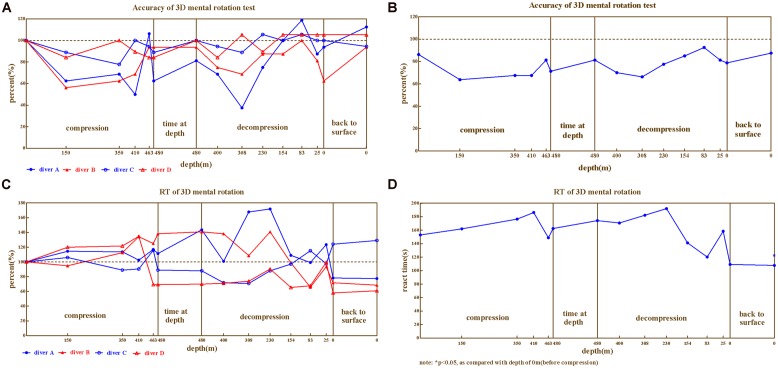
**The results from 3D mental rotation. (A)** Correct rate for four divers, **(B)** Correct rate on Average, **(C)** Reaction time for four divers, **(D)** Reaction time on average.

In 3D mental rotation, the correct rates (**Figures 3A,B**) and reaction times (**Figures 3C,D**) of the four divers exhibited considerable impairment and large variability across individual, with reaction times ranging 58.0%∼226.0% and correct rates 37.5%∼118.8%. Correct rates of all the four divers declined as compression processed, while diver C and D were less impaired than A and B (ranged 77.8%∼105.6%, 84.2%∼105.3% vs. 37.5%∼118.8%, 56.3%∼100.0%, in compared to baseline). There was enormous variability on reaction times of four divers. Only small impairment was detected on diver C during the compression and decompression but that became a little greater after the dive, on the contrary, the reaction times of diver A and B increased throughout the dive until they came back to the surface. The pattern of reaction time for diver D was different from above, whose reaction time increased as compression was performed but restored since the depth of 463 in compression.

Repeated variance analyses revealed that the depth had a significant effect on reaction time [*F*_(14,42)_ = 3.105, *P* < 0.01, $\eta_{p}^{2}$ = 0.509], as well as on correct rate [*F*_(14,42)_ = 3.129, *P* < 0.01, $\eta_{p}^{2}$ = 0.511]. LSD *post hoc* test revealed that during the compression phase, the reaction time continuously increased and the correct rate decreased until 410 m for reaction time (*P* > 0.05 in compared to predive) and 150 m for correct rate (*P* > 0.05, when compared to predive).

We further compared the 3D mental rotation ability across different phases of the experiment (predive, compression, stay, decompression, 3 days, and 1 month after the dive; **Table 2**). Repeated variance analyses revealed that the phase of the experiment had a significant effect on the reaction time [*F*_(5,15)_ = 12.10, *P* < 0.01, $\eta_{p}^{2}$ = 0.801], but not on the correct rate [*F*_(5,15)_ = 6.09, *P* > 0.05, $\eta_{p}^{2}$ = 0.465]. LSD *post hoc* test revealed that the reaction times of the four divers slightly increased in the compression/stay/decompression phases (when compared to the baseline, *P* > 0.05) but decreased significantly after the dive (*P* < 0.01 when compared to baseline).

**Table 2:** 3D mental rotation ability across different phases of the experiment (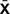 ± SD, *n* = 4).

Phase	Reaction time(s)	Correct rate(%)
Predive	153.00 ± 21.76	86.25 ± 7.50
Compression	168.38 ± 24.27	70.00 ± 15.24
Stay	160.69 ± 22.48	76.25 ± 13.62
Decompression	160.83 ± 18.87	78.75 ± 15.07
3 days after	109.25 ± 14.06	78.75 ± 21.75
1 month after	107.75 ± 11.79	87.50 ± 10.41

#### Spatial Memory

**FIGURE 4:**
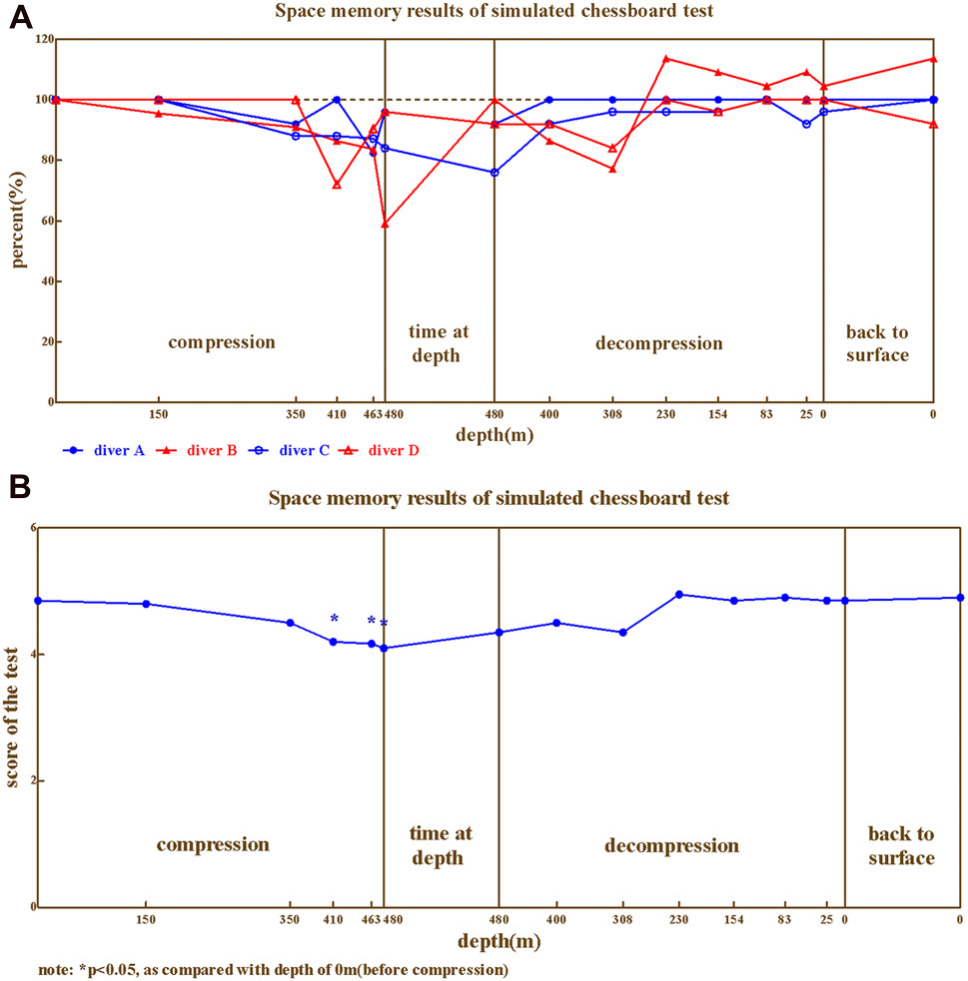
**The results from spatial memory. (A)** Four divers, **(B)** Average.

The spatial memory ability (**Figures 4A,B**) of the four divers exhibited large variability across individuals. The performance of diver A appears to be stable compared to baseline, except 350–480 m in compression and stay phase ranging from 82.4%∼96.0%. Greater impairment was observed on diver B and C, which decreased as compression processed until 480 m (59.1% for diver B and 76.0% for diver C, compared to baseline). While, the worst performance for diver D was at the depth of 410 m in compression (72.0% compared to baseline).

Repeated variance analyses and LSD *post hoc* test revealed that the depth has a significant effect on spatial memory [*F*_(14,42)_ = 2.809, *P* < 0.01, $\eta_{p}^{2}$ = 0.484], which was significant impaired during 410–480 m in compression and stay phase (*P* < 0.05). During the compression phase, the spatial memory continuously decreased until 480 m (*P* < 0.05 in compared to baseline); while spatial memory returned to baseline as decompression started (*P* > 0.05 when compared to predive).

We further compared the spatial memory ability across different phases of the experiment (Predive, compression, stay, decompression, 3 days and 1 month after the dive; **Table 3**). Repeated variance analyses revealed that the phase of the experiment has a significant effect on spatial memory [*F*_(5,15)_ = 5.19, *P* < 0.01, $\eta_{p}^{2}$ = 0.634]. LSD *post hoc* test revealed that the spatial memory of the four divers slightly decreased in the compression phases (when compared to the baseline, *P* > 0.05), and significantly decreased in the stay phase (*P* < 0.05 to baseline), and returned to baseline level during the decompression phase (*P* > 0.05 to baseline).

**Table 3:** Spatial memory ability across different phases of the experiment (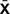 ± SD, *n* = 4).

Phase	Test results (*n*)
Predive	4.85 ± 0.30
Compression	4.42 ± 0.34
Stay	4.23 ± 0.59
Decompression	4.73 ± 0.25
3 days after	4.85 ± 0.19
1 month after	4.90 ± 0.20

### Performance Efficacy

#### Grip Strength

**FIGURE 5:**
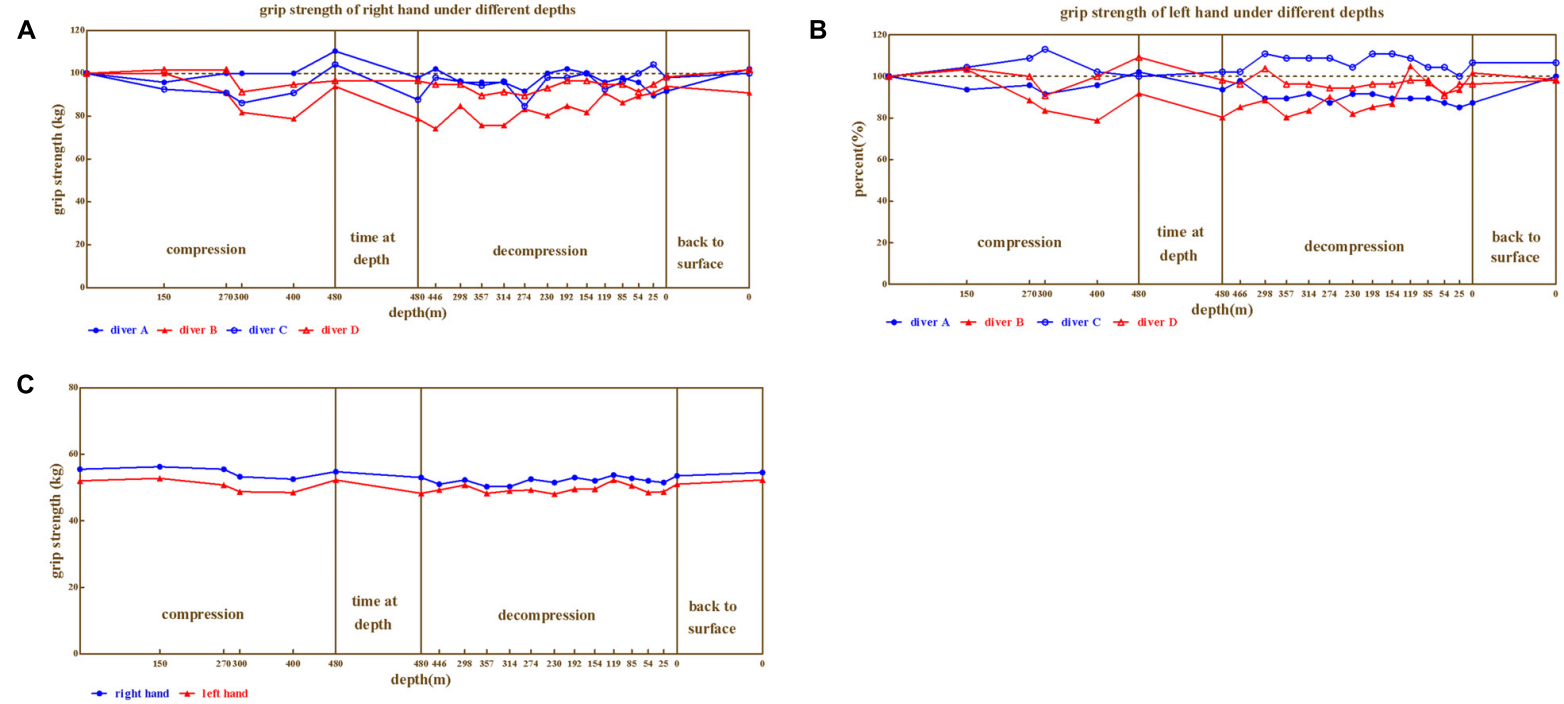
**The results from grip strength. (A)** Right hand for divers, **(B)** Left hand for four divers, **(C)** Left and right hand on average, respectively.

The right hand (**Figure 5A**) and left hand (**Figure 5B**) grip strength also showed individual variability (ranged 74.24%∼113.04% of baseline). Both left-hand and right-hand grip strength of diver B were associated with the depth, which declined as compression processed and restored during decompression. While, slight impairment was detected for diver D. Interestingly, left-hand grip strength of diver C was even higher than baseline. Furthermore, the best performances of right-hand grip strength for diver A and C were observed at the depth of 480 m, as well as best right-hand performance for diver D (110.4, 104.2, and 109.3% respectively, when compared to baseline).

Repeated variance analyses revealed that the depth had no significant effect on right hand grip strength [*F*_(20,60)_ = 1.029, *P* > 0.05, $\eta_{p}^{2}$ = 0.255], as well as the left hand grip strength [*F*_(20,60)_ = 1.078, *P* > 0.05, $\eta_{p}^{2}$ = 0.264]. In general (**Figure 5C**), the depth above 230 m are associated with lower grip strength.

We further compared the grip strength across different phases of the experiment (Predive, compression, stay, decompression, 3 days, and 1 month after the dive; **Table 4**). Repeated variance analyses revealed that the phase of the experiment has no significant effect on both right [*F*_(5,15)_ = 0.83, *P* > 0.05, $\eta_{p}^{2}$ = 0.217] and left hand [*F*_(5,15)_ = 0.63, *P* > 0.05, $\eta_{p}^{2}$ = 0.173] grip strength. Both left and right hand grip slightly decreased at stay and decompression phase.

**Table 4:** Grip strength of left and right hand across different phases of the experiment (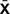 ± SD, *n* = 4).

Phase	Right hand	Left hand
Predive	55.50 ± 8.23	52.00 ± 6.98
Compression	54.38 ± 5.32	50.19 ± 5.23
Stay	53.88 ± 4.94	50.25 ± 5.23
Decompression	51.90 ± 4.20	49.46 ± 5.21
3 days after	53.50 ± 7.77	51.00 ± 8.68
1 month after	54.50 ± 5.80	52.25 ± 5.74

#### Hand–Eye Coordination

**FIGURE 6:**
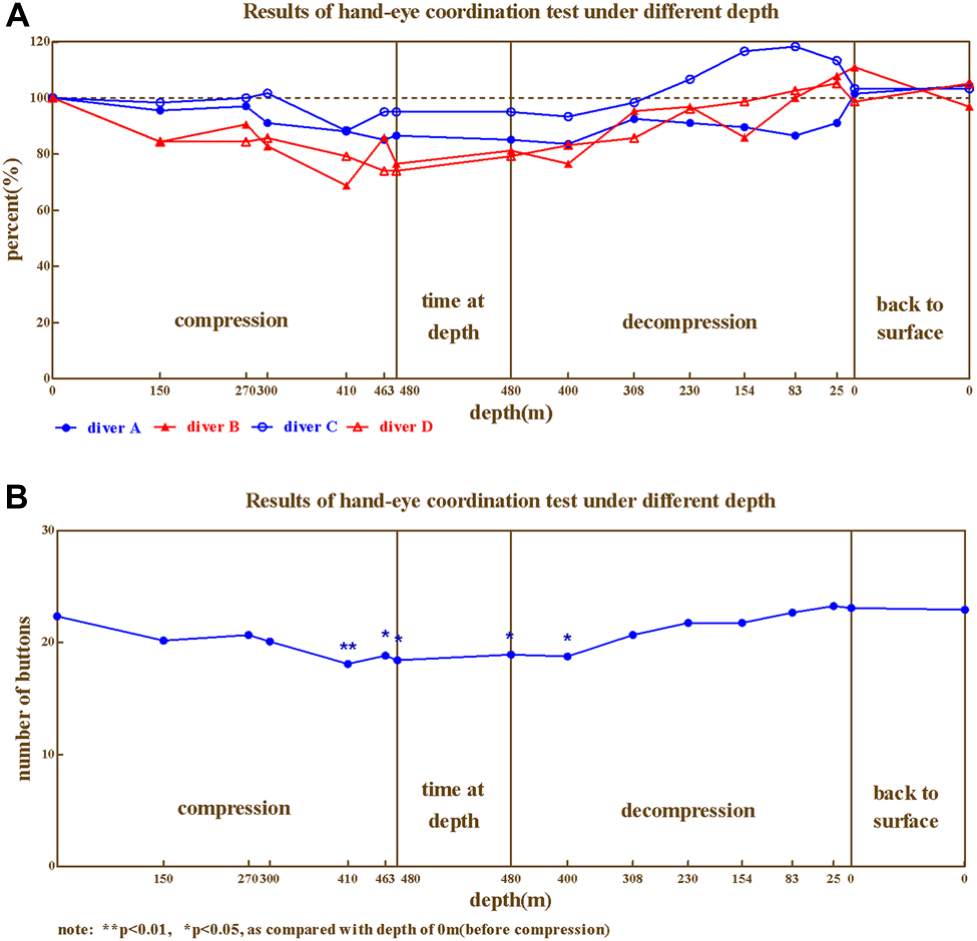
**The results from hand–eye coordination. (A)** Four divers, **(B)** Average.

The hand–eye coordination ability of the four divers during compression showed similar trends of change (**Figures 6A,B**), ranged 68.75%∼118.33%. The lowest values were at 400–480 m.

Repeated variance analyses revealed that the depth had a significant effect on hand–eye coordination ability [*F*_(15,45)_ = 6.50, *P* < 0.05, $\eta_{p}^{2}$ = 0.684]. LSD *post hoc* test revealed that During the compression phase, the hand–eye coordination ability showed no significant changes at 150, 270, and 300 m, and decreased at 410, 463 m (*P* < 0.05 when compared to baseline); while during the decompression phase the hand–eye coordination ability restored at 308 m (*P* > 0.05 when compared to predive).

We further investigated hand–eye coordination ability across different phases of the experiment (Predive, compression, stay, decompression, 3 days, and 1 month after the dive; **Table 5**). Repeated variance analyses revealed that the phase of the experiment had a significant effect on hand–eye coordination ability [*F*_(5,15)_ = 8.113, *P* < 0.01, $\eta_{p}^{2}$ = 0.730]. LSD *post hoc* test revealed that the hand–eye coordination ability of the four divers slightly decreased in the compression phase (when compared to the baseline, *P* > 0.05), and significantly decreased in the stay phase (*P* < 0.05 to predive), and returned to baseline level during the decompression phase (*P* > 0.05 when compared to predive).

**Table 5:** Hand–eye coordination ability across different phases of the experiment (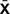 ± SD, *n* = 4).

Phase	Test results (*n*)
Predive	22.33 ± 2.42
Compression	19.57 ± 1.78
Stay	18.67 ± 1.25
Decompression	21.47 ± 2.63
3 days after	23.08 ± 1.95
1 month after	22.92 ± 3.00

## Discussion

Chronic psychological or physical stress is known to induced physiological changes in the brain, impairments in cognition, sensation of the external world, and general physiological alterations (Hou et al., 2014, 2015; Tian et al., 2014; Yuan and Hou, 2015; Yuan et al., 2015). Extreme environment stress has been known to induce changes in both the body and mind, as the case of deep water diving with high atmosphere pressure (Curley et al., 1979; Biersner et al., 1984; Nagashima et al., 2002; Hirayanagi et al., 2003; Brubakk et al., 2014). In present study with simulated helium–oxygen saturation diving experiment, we found that the correct rate of 2D mental rotation was relatively impaired under the high atmosphere pressure, while the 3D mental rotation ability was significantly impaired, suggesting that 3D task is more vulnerable to the depth of diving. In addition, the reaction time of 2D and 3D mental rotation increased with the depth. The 410 m depth seems to be the changing point between reaction time increase and becoming stable, potentially due to the adaption effect. This is in line with previous study showing that divers react to high pressure as slowing down their working speed, in order to maintain the working efficacy (30 m, air diving; Petri, 2003). It is also realized that making a mistake in performance will result in serious consequences in diving task (Logie and Baddeley, 1983), and therefore the working speed is relatively less important.

In addition, the spatial memory was significantly impaired with compression between 410 and 480 m, showing large individual variability. Previous study also revealed decreased visual spatial ability in diving at 300–540 m (Lewis and Baddeley, 1981). Notably, the parietal lobe is known to be involved in both spatial cognition (Culham and Valyear, 2006) and mental rotation (Harris and Miniussi, 2003). Whether the high atmosphere pressure leads to dysfunction of parietal lobe is yet to be investigated in future studies.

The mental abilities are critical elements determining the task performance efficacy. We chose the grip strength and hand–eye coordination ability as the two measurements as previously described (Lewis and Baddeley, 1981; Logie and Baddeley, 1983; Petri, 2003). We found that the grip strength is mildly affected. In previous study with open sea dive, the high pressure led to increased tremor, decreased grip strength, decreased speed of steeping, and slower spontaneous reaction (Vaernes et al., 1987). It is also found that during the compression, the EEG θ wave significantly increased, while α, β_1_, and β_2_ activity decreased (Bennett and Towse, 1971), which might explain the changes in grip strength.

The hand–eye coordination was clearly impaired by the high pressure environment, which declined continuously to 480 m. In one study, it was found that the finger flexibility decreased by 2% at 180 m and 10% at 450 m (Rostain et al., 1983); while in another study with helium–oxygen saturation diving, the authors reported no changes in finger flexibility at 360 m (Hamilton, 1976). We believe that this might be due to the large individual differences. Three-hundred meter might be the turning point of tremor induction, and therefore the decrease in hand–eye coordination ability.

In summary, we report that the mental abilities and performance efficacy are significantly impaired during deep water helium–oxygen saturation diving.

## Conflict of Interest Statement

The authors declare that the research was conducted in the absence of any commercial or financial relationships that could be construed as a potential conflict of interest.
